# Levothyroxine Therapy in Elderly Patients With Hypothyroidism

**DOI:** 10.3389/fendo.2021.641560

**Published:** 2021-03-12

**Authors:** Grigoris Effraimidis, Torquil Watt, Ulla Feldt-Rasmussen

**Affiliations:** ^1^ Department of Medical Endocrinology and Metabolism, Rigshospitalet, Copenhagen University Hospital, Copenhagen, Denmark; ^2^ Department of Clinical Medicine, Faculty of Health and Medical Sciences, University of Copenhagen, Copenhagen, Denmark; ^3^ Department of Internal Medicine, Endocrine Section, Copenhagen University Hospital Herlev Gentofte, Copenhagen, Denmark

**Keywords:** levothyroxine, thyroid treatment, elderly, hypothyroidism, older adults, thyroid

## Abstract

Levothyroxine (L-T4) treatment of overt hypothyroidism can be more challenging in elderly compared to young patients. The elderly population is growing, and increasing incidence and prevalence of hypothyroidism with age are observed globally. Elderly people have more comorbidities compared to young patients, complicating correct diagnosis and management of hypothyroidism. Most importantly, cardiovascular complications compromise the usual start dosage and upward titration of L-T4 due to higher risk of decompensating cardiac ischemia and -function. It therefore takes more effort and care from the clinician, and the maintenance dose may have to be lower in order to avoid a cardiac incidence. On the other hand, L-T4 has a beneficial effect on cardiac function by increasing performance. The clinical challenge should not prevent treating with L-T4 should the patient develop e.g., cardiac ischemia. The endocrinologist is obliged to collaborate with the cardiologist on prophylactic cardiac measures by invasive cardiac surgery or medical therapy against cardiac ischemic angina. This usually allows subsequent successful treatment. Management of mild (subclinical) hypothyroidism is even more complex. Prevalent comorbidities in the elderly complicate correct diagnosis, since many concomitant morbidities can result in non-thyroidal illness, resembling mild hypothyroidism both clinically and biochemically. The diagnosis is further complicated as methods for measuring thyroid function (thyrotropin and thyroxine) vary immensely according to methodology and background population. It is thus imperative to ensure a correct diagnosis by etiology (e.g., autoimmunity) before deciding to treat. Even then, there is controversy regarding whether or not treatment of such mild forms of hypothyroidism in elderly will improve mortality, morbidity, and quality of life. This should be studied in large cohorts of patients in long-term placebo-controlled trials with clinically relevant outcomes. Other cases of hypothyroidism, e.g., medications, iodine overload or hypothalamus-pituitary-hypothyroidism, each pose specific challenges to management of hypothyroidism; these cases are also more frequent in the elderly. Finally, adherence to treatment is generally challenging. This is also the case in elderly patients, which may necessitate measuring thyroid hormones at individually tailored intervals, which is important to avoid over-treatment with increased risk of cardiac morbidity and mortality, osteoporosis, cognitive dysfunction, and muscle deficiency.

## Introduction

According to World Population Prospects 2019 (United Nations, 2019), the proportion of the population aged 65 years or over has risen from 6% in 1990 to 9% in 2019 and it is expected to rise further to 16% by 2050 (1). The average life expectancy has undergone the fastest rise between 2000 and 2016 since the 1960s (2) and survival beyond age 65 is globally improving, as a person aged 65 years in 2015–2020 could expect to live, on average, an additional 17 years. Unsurprisingly, this demographic progress is accompanied by increasing prevalence of multiple chronic diseases, increased (multi)morbidity and disability and consequently polypharmacy with higher risk of drug interactions and adverse effects (3).

Hypothyroidism is a common condition caused by thyroid hormone deficiency. Most commonly, the pathology is within the thyroid gland and hence termed primary hypothyroidism, which biochemically is characterized by increased serum thyroid-stimulating hormone (TSH) concentrations. It is subdivided depending on the circulating free thyroxine (fT4) concentrations into overt hypothyroidism when fT4 was lower than the population-based reference range and subclinical hypothyroidism, when fT4 was within the population-based reference range (4). The latter is in turn subdivided into grade 1 (mild) subclinical hypothyroidism, when TSH is between the upper normal limit and 10 mU/l, and grade 2 (severe) subclinical hypothyroidism when TSH is ≥10 mU/l (5).

The prevalence of overt hypothyroidism in the general population ranges from 0.1 to 2% (6–9), while the prevalence of subclinical hypothyroidism is much higher varying from 4 to 10% (6, 8, 10, 11). The prevalence of hypothyroidism increases with age and subclinical hypothyroidism affects up to 15% of adults 65 years of age or older, when non-age-specific TSH reference ranges are used (9, 12–14). Spontaneous hypothyroidism is about 10 times more prevalent in women compared to men (15). By each age decade the proportion of women with increased serum TSH concentrations was higher compared with the one of men in the Colorado Thyroid Disease Prevalence study (9).

## Challenges in the Diagnosis of Hypothyroidism in the Elderly

Hypothyroid symptoms are non-specific and vary among patients, especially in the setting of subclinical hypothyroidism. The same symptoms are also quite common in euthyroid individuals and thus often overlap with the symptoms developed in patients with hypothyroidism (9). Although hypothyroidism-associated symptoms may indicate and identify hypothyroidism in most young patients, this is rarely the case in the elderly (16). Conversely, actual hypothyroidism causing tiredness, sleep disorders, depression, lack of concentration and amnesia in old individuals may be overlooked as these symptoms can be interpreted as normal age-related changes by both physicians and patients.

Convincing evidence during the last decades has shown an age-dependent shift in TSH distribution towards higher concentrations with increasing age. In the NHANES III study, median TSH concentrations progressively increased with age and the 97.5th percentiles were considerably higher in the >70 years old reference population without thyroid antibodies (97.5th percentile TSH in the reference population: total 4.1 mU/l; 70–79 yo 5.9 mIU/l; >80 yo 7.5 mU/l) (11). Similar results were obtained in other populations, such as in Scotland (97.5th percentile TSH 4.0, 5.5 and 5.9mU/l for 31–40 yo, 80–90 yo and >90 yo, respectively), in Ashkenazi Jews (4.6 and 7.2 mU/l at a median age of 72 and 98 years, respectively), in Americans (5.2 and 6.8 mU/l for 20–29 yo and >80 yo, respectively) and in Chinese (6.6 and 8.9 mU/l in <65 yo and ≥65 yo, respectively) (17–20). Iodine intake and thyroid autoimmunity are important factors to consider when looking at the epidemiology of hypothyroidism across ages and in any populations (21–23). Even a cautious iodine fortification in a population can change the incidences rather dramatically (21, 24, 25). Autoimmune hypothyroidism is the most common cause of hypothyroidism at all ages and the prevalence of thyroid autoimmunity increases with aging (23, 26, 27).

Nevertheless, the higher prevalence of thyroid autoimmunity in the older population can only partially explain the higher TSH concentrations with increasing age. Thus, among the thyroid antibody negative persons from the NHANES III study there was an age-dependent increase in TSH concentrations and longitudinal data have suggested that TSH generally increases over time and with age in the same subject especially in older individuals (28, 29). The interindividual age-dependent TSH rise was not associated with a decline in fT4 nor with increased mortality, suggesting that the TSH increment might reflect an age-related alteration in the TSH set point and/or reduced TSH bioactivity and/or reduced sensitivity of the thyroid gland to TSH rather than occult thyroid disease (30). When age-specific reference ranges were employed in the NHANES III study, 70% of the >80 yo group was reclassified as having normal for their age TSH rather than high TSH based on the reference range of the general population (>4.5 mU/l) (31). In addition, when the age-adjusted TSH reference ranges were used, no association between thyroid function and quality of life, mood, and cognition at baseline nor over the 5–8 years of follow-up in community-dwelling older men was found (32).

Longevity was associated with higher TSH concentrations in the Ashkenazi population (19) and confirmed by two Dutch studies (the Leiden 85-Plus Study and the Leiden Longevity Study) (33–35). Men and women aged 85 years with abnormally high TSH concentrations according to the general reference range for younger people and abnormally low concentrations of fT4 had the lowest mortality rate during the 3.7-yr follow-up (33). Analysis of combined data from nonagenarians from long-lived families from the Leiden Longevity Study and nonagenarians from the general population from the Leiden 85-Plus Study revealed an association between risk of mortality and lower fT4, higher free thyronine (fT3) and higher fT3/fT4 ratio, but not with higher TSH (36). The lower basal metabolic rate due to lower fT4 activity has been proposed as a possible explanation for the association between TSH and longevity (35).

A drug review process should always be conducted before the diagnosis of hypothyroidism. This is especially important for the older people as they very often present with increased (multi)morbidity and excess amount of prescribed medications. A number of medications can affect the thyroid function tests not only by interfering with the synthesis, transport, and metabolism of TSH and thyroid hormones but also by interfering with thyroid function immunoassays (30, 37–40) (**Table 1**).

**Table 1:** Drugs with an increased likelihood of inducing thyroid dysfunction.

Inhibit thyroidhormone production	Alter extra-thyroidal metabolismof thyroid hormone	Alter T4/T3 bindingto plasma proteins	Induction of thyroiditis	Affection of TSH secretion	Impairing absorption of oral T4
Antithyroid drugs	Propylthiouracil	Estrogen	Amiodarone	Lithium	Aluminum hydroxide
Amiodarone	Glucocorticoids	Heroin	Interleukin-2	Dopamine Receptor Blockers	Ferrous Sulfate
Lithium	Propranolol	Methadone	Interferon-α	L-Dopa Inhibitors	Cholestyramine
Iodide (large doses)	Amiodarone	Clofibrate	Interferon-β	Cimetidine	Calcium Carbonate
Iodine-containing contrast media	Iodine-containing contrast media	5-Fluorouracil	γ-Interferon	Clomifene	Calcium Citrate
	Carbamazepine	Perphenazine	Sunitinib	Thyroid Hormone	Calcium Acetate
	Barbiturates	Glucocorticoids	Monoclonal antibody therapy (the check point inhibitors: Nivulomab, Pembrolizumab, Ipilimimab)	Dopamine	Iron Sulfate
	Rifampicin	Androgens		L-Dopa	Colestipol
	Phenytoin	L-Asparaginase		Glucocorticoids	Sucralfate
	Sertralin	Nicotinic Acid		Growth Hormone	Soya preparations
		Furosemid		Somatostatin	Kayexalate
**Other**		Salicylates		Octreotide	Ciprofloxacin
Thalidomide		Phenytoin			Sevelamer
Lenalidomide		Fenclofenac			Proton pump inhibitors
Chemotherapy for sarcoma		Heparin			

**Figure 1:**
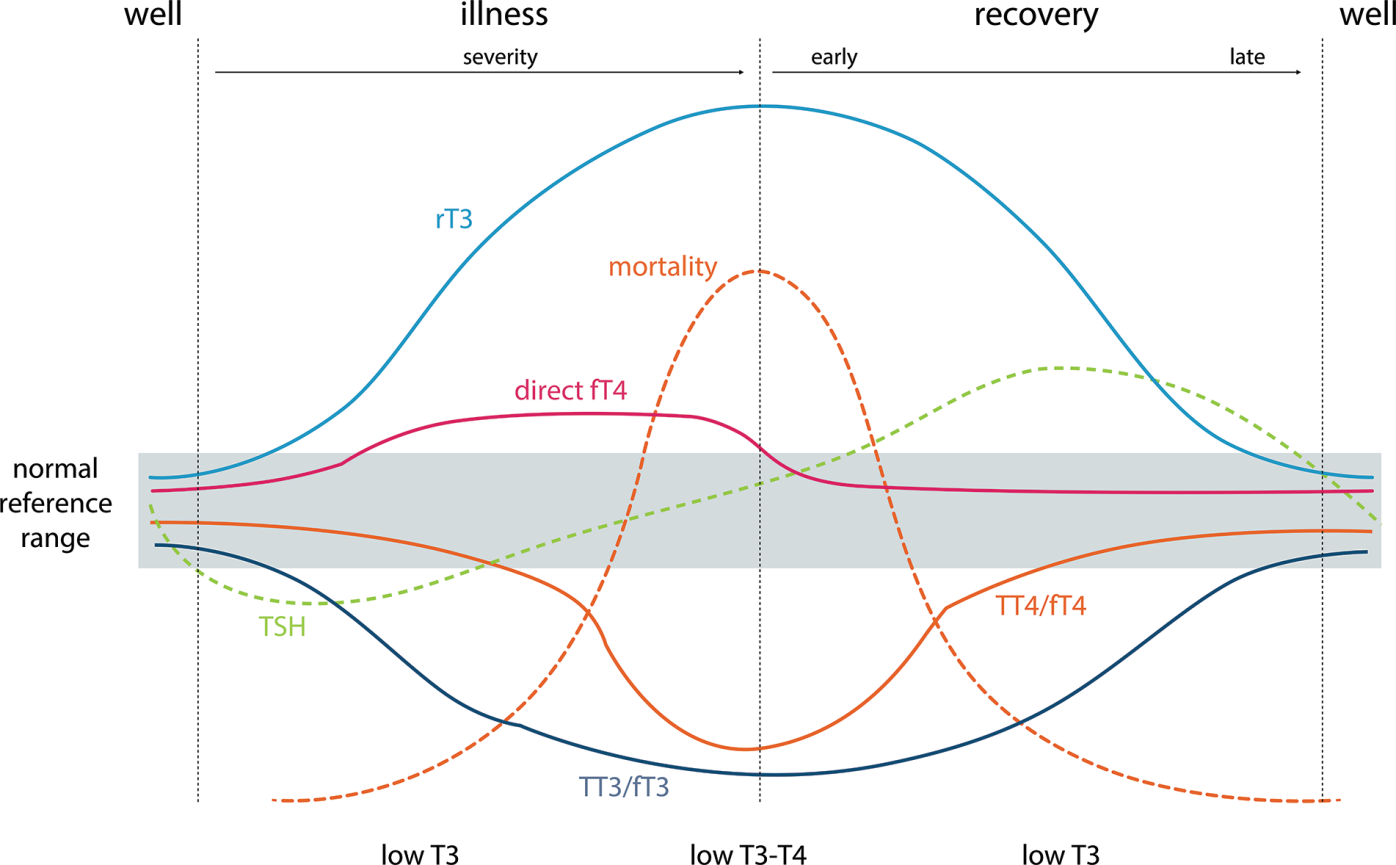
Typical changes in thyroid function tests during the development of and recovery from nonthyroidal illness and their relationship to mortality. TSH, thyrotropin; TT3/TT4, total thyroid hormones; FT3/FT4, measured free thyroid hormone estimates; direct FT4, direct measurement of free T4 by dialysis or ultrafiltration = “True free T4”; rT3: reverse T3. Adapted from Demers and Spencer eds. (44).

The much more prevalent comorbidities in the elderly may result in alterations in thyroid function as part of the euthyroid sick syndrome. Although the euthyroid sick syndrome classically presents in critically ill patients  (41, 42), it can also develop in the setting of common chronic conditions such as heart, kidney, liver disease, diabetes, major depression, as well as low caloric intake  (43). The biochemical hallmark of the euthyroid sick syndrome is very low T3 concentrations in the presence of normal or slightly decreased TSH (**Figure 1**) (**Table 2**) (41, 42), and thus a T3 measurement should be performed if euthyroid sick syndrome is suspected. On progression a low T4 is usually observed as well, while TSH is often elevated in the restoration phase (41, 42). To date, treatment with L-T4 is not indicated in this situation, with the exception of patients in whom pre-existing primary hypothyroidism and euthyroid sick syndrome co-exist.

**Table 2:** Some situations in which serum TSH alone can give a false or uncertain indication of thyroid status in elderly people compared to the normal reference interval in young persons.

Condition	TSH	fT4	fT3
**Primary abnormality/change of TSH secretion**			
Increasing age	H	N	N
Pituitary-hypothalamic abnormality	L-N	L	L
Central TSH excess	N-H	H	H
**Hyperthyroidism**			
T3 toxicosis	S	N	H
Subclinical	S	N	N
Early Treatment with antithyroid drugs	S	H-N-L	H-N-L
TSH assay artefact	L-N-H	H	H
**Hypothyroidism**			
Subclinical	H	N	N
Early Treatment with levothyroxine	H	L-N	L-N
TSH assay artefact	H	N	N
**Thyroid hormone resistance**	N -H	H	H
**Euthyroid Sick syndrome and recovery**	L-N-H	L-N	L
**Medications**			
Dopamine	L	N	N
Glucocorticoids	L	N	L-N
Amiodarone (acute)	H	N-H	L

N, normal; L, low; H, high; S, suppressed.

## Indication for Levothyroxine Treatment of Hypothyroidism in Elderly

The indication for L-T4 treatment of overt hypothyroidism is similar in young and elderly hypothyroid patients. However, more caution must be paid to a variety of the complicating factors that are more prevalent with increasing age. Firstly, a correct diagnosis is more complicated due to the many possible comorbidities that can give rise to a falsely elevated serum TSH concentration even above 10 mU/L as required for the diagnosis of overt hypothyroidism due to (a) recovery from a lowered serum TSH seen in severe nonthyroidal illnesses (**Figure 1**), (b) recovery after different types of destructive thyroiditis (subacute, autoimmune, symptomless autoimmune thyroiditis), (c) medications such as lithium (treatment for bipolar maniodepressive psychosis), the antiarrythmic drug amiodarone, and glucocorticoids, which can inhibit thyroid hormone synthesis and metabolism and may cause transient reversible elevation of serum TSH (d) immune modulating drugs for treatment of other autoimmune diseases and cancers with ability to induce a transient autoimmune type of thyroiditis as in (b) (39), and (e) presence of big TSH or heterophile antibodies in the patient’s serum (**Table 1**, **Table 2**).

A complimentary measurement of fT4 estimate must be done in all cases (either by total T4 combined with a measure of binding proteins or by one of the fT4 clinical biochemical platforms used in laboratories worldwide), while measurement of serum T3 is not recommended for the treatment indication of hypothyroidism (15); T3 may be relevant for identifying euthyroid sick syndrome, though. T4 measurements can give rise to falsely low concentrations in case of molecular changes in the thyroid hormone binding proteins in serum: thyroxine binding globulin, transthyretin, or albumin or through their binding affinity for T4. Circulating T3- or T4- binding autoantibodies can cause methodological artefacts in both total and free measurements of T4 (40, 45–47) as can antibodies against assay reagents (e.g., antiruthenium, antistreptavidin, or antibiotin). High dose biotin ingestion by the patient has also been shown to result in serious distortion of analyte- and platformspecific assay results, and is now a frequent cause of false results due to the current popularity of biotin ingestion for skin and hair beauty products (47). To increase the likelihood of true hypothyroidism and not only a biochemical quirk it is helpful to search for the etiology of the disease, such as presence of anti-peroxidase antibodies in thyroid autoimmunity, history of previous surgery or radioiodine therapy or other important causes.

When the diagnosis is secured eventually by reanalysis of samples drawn after 3–6 months and/or testing in a different laboratory using different measurement methodology, the clinician will be faced with the challenge of assessing the current cardiac situation of the patient. Hypothyroidism has a profoundly negative effect on cardiac performance (**Table 3**) which results in low exercise performance, and more prominently so in elderly patients. This is particularly the case in patients with a pre-existing heart failure, which should always be considered a possibility in the evaluation of older patients with hypothyroidism (48, 49). Even in asymptomatic individuals it is therefore pertinent to perform a very rigorous assessment of elderly hypothyroid patients before commencement of L-T4 therapy in order to avoid provoking cardiac ischemia and/or insufficiency by increasing the resting metabolic rate. In case of very high age and/or suspicion of a cardiac condition the patient may require a stress test or coronary angiography to aid in the risk assessment. In case of any cardiac issues it is wise to consult a cardiologist also to discuss possible relevant prophylactic treatment options, to open the vessels surgically in case of stenosis or by antianginous medications (50). It is also sometimes prudent to start levothyroxine therapy in patients with cardiac conditions during hospitalization and monitoring of cardiac rhythm and function.

**Table 3:** Hemodynamic changes in hypothyroidism.

Myocardial contractility	↓
Peripheral vascular resistance	↑
Circulation time	↑
Diastolic blood pressure	↑
Arterial stiffness	↑
Left ventricular stroke volume	↓
Left ventricular systolic function	↓
Left ventricular diastolic function	↓
Cardiac output	↓
Cardiac index	↓
Exercise tolerance	↓

It is important to realize that normal thyroid function and thus also L-T4 therapy of overt hypothyroidism is eventually beneficial for cardiac function (**Table 4**) (51), so it is clinically imperative to make an effort to persuade the patient to comply with the treatment even if there are obstacles to starting the therapy.

**Table 4:** Treatment of hypothyroidism with levothyroxine—cardiac concerns and effects on these risk factors.

Concerns	Effects
Cardiac insufficiency	Normalizes cardiac output
Ischemia and angina pectoris	Normalizes left ventricular contractile performance
Tachyarrhythmias	Lowers diastolic blood pressure
Pericardial effusion	Decreases serum cholesterol
High output failure without preexisting heart disease	Normalizes diastolic dysfunction
	Normalizes endothelial dysfunction

Both diagnosing and decision of treatment or not are much more difficult in patients with mild or subclinical hypothyroidism in the elderly for a variety of reasons (5). The diagnosis is more challenging than that of overt hypothyroidism due partly to all of the above mentioned complicating and confounding factors being even less clear in discriminating between normal thyroid function and mild hypothyroidism in elderly persons compared with young ones: (a) The upper reference limit of serum TSH concentrations in healthy normal elderly people is highly variable with age and not studied in populations at large, nor by different laboratory platforms. Ideally, each laboratory should perform its own age specific population specific reference interval across the age range including centenarians in order to diagnose the condition correctly. This, however, rarely happens. The upper limit of serum TSH in the older population can be up to 7.5–8.8 mU/L which does not leave much space up to 10 mU/L when also considering the method related imprecision of serum TSH measurements (**Table 2**). (b) Symptoms are milder and less discriminative in the elderly. (c) It puts a strong responsibility on clinicians to make sure to diagnose correctly and avoid misclassification resulting in incorrect commencement of L-T4 (**Table 2**). This is so much more important because the risk of overdosing is high in the elderly and treatment with L-T4 of persons with normal thyroid function but with variable symptoms that might be due to hypothyroidism is strongly advised against (4, 15). (d) The presence of thyroid antibodies, which is associated with an increased risk of progression from subclinical to overt hypothyroidism, suggests a closer monitoring of thyroid function in subjects with thyroid antibodies (27, 52). On the contrary, normalization of TSH occurs more often in thyroid antibodies negative subjects. (e) L-T4 therapy is more challenging in elderly patients with many comorbidities and multipharmacy with drugs that can also influence the absorption of T4 (53) (**Tables 1** and **2**); not to forget difficult compliance in patients receiving a multitude of drugs.

The frailty status is another important factor to consider before initiation of LT4 treatment of elderly people with subclinical hypothyroidism. The frail elderly are vulnerable to drugs side effects, overtreatment and poor compliance (54). These considerations as well as a possible positive effect of thyroid autoimmunity on frailty status (55) suggest a conservative wait-and-see approach for frail older patients even in the presence of thyroid autoimmunity (54).

Some of these challenges can be overcome by getting a good overview of the patient’s concomitant diseases, or eventually look for other likely candidates as explanation for the patient’s complaints such as presence of other autoimmune diseases, particularly those that might compromise T4 absorption such as pernicious anemia, coeliac disease and ulcerative colitis (56), or by prescribing other T4 formulations such as an easily absorbable gel capsule (57, 58).

## Titration of Levothyroxine Therapy in Elderly Patients and Monitoring of Effect

Due to the vague symptoms of subclinical hypothyroidism also in the elderly, the diagnosis is often suggested by incidental discovery of a high TSH within a package of blood measurements in persons showing up at the general practitioner for being tired. Anyway, if deciding on performing a therapeutic trial together with the patient, proper treatment monitoring and particularly avoiding overdosing is extremely important not to put the patient at risk.

Once a patient-clinician agreement on initiating levothyroxine treatment has been reached, three main issues are particularly relevant in the elderly patient, in order to ensure appropriate treatment: Is cardiac comorbidity present? How should treatment be initiated? What is the treatment target to aim for?

In case cardiac co-morbidity has been ruled out, possibly in collaboration with a cardiology expert, it seems safe to start similarly as in younger patients  (59); nonetheless, most clinicians start at lower doses and up-titrate at a slower pace, acknowledging the general frailty of this age-group.

Lacking good evidence the treatment target is mostly empirically based and could be either (a) TSH (ideally related to an age specific reference range), (b) other biochemical and clinical indices of thyroid function or (c) patient-experienced variables, e.g., thyroid-related patient-reported outcomes (PRO). Usually, serum TSH concentrations are aimed at a higher TSH than in younger patients, respecting the possibly better health outcomes associated with higher TSH in old age (4, 53). Similarly, fT4 is aimed at a concentration in the lower half of the reference range. However, no trials have substantiated this approach, since no blinded randomized placebo-controlled trials of L-T4 treatment in elderly patients with hypothyroidism comparing different TSH targets have been published.

Blood-lipids are frequently monitored during L-T4 therapy as indication of treatment effect. However, there is no reliable laboratory index of peripheral thyroid hormone action, but some tests (27, 60), including sex steroid- binding globulin, serum ferritin, serum angiotensin- converting enzyme, as well as oxygen consumption (resting energy expenditure), systolic time interval, and cardiac contractility (61, 62), may be useful in rare unclear cases of following the individual response in situations of suspected thyroid hormone resistance or during long-term suppressive therapy with T4.

Due to its long history, introduction of L-T4 treatment for overt hypothyroidism was not preceded by modern randomized clinical trials  (63) and thus data on patient-reported outcome of treatment mostly rely on observational studies. Generally, levothyroxine treatment has been shown to improve QoL (including symptoms) in patients with hypothyroidism  (62). However, since symptoms and thus the patient-experienced manifestations of hypothyroidism are vaguer among the elderly (16), effects observed in younger populations cannot unquestionably be extrapolated to older ones. The limited symptomatology implies smaller patient-experienced treatment effects, which may also decrease motivation for treatment initiation and adherence in individual patients.

The fewer symptoms in older patients will also impede recognition of a potential treatment effect in randomized clinical trials. This may particularly be the case in patients with subclinical hypothyroidism and may have influenced the negative findings in previous randomized clinical trials (64), reviewed by Feller et al.  (65). However, secondary analyses in patients with higher symptom loads from the largest trial among elderly patients corroborated the lack of patient-experienced effect (66). Regrettably, no counterpart to the above mentioned randomized clinical trial by Stott et al., has been conducted in patients with overt disease; even well-designed descriptive longitudinal studies exploring the effect of L-T4 treatment on quality of life among elderly are lacking (14).

Apart from titrating L-T4 to an appropriate biochemical target, a classical patient-physician encounter in terms of the physician inquiring about symptoms of over-replacement as part of a clinical interview is paramount for proper management. To date, no studies evaluating a systematic approach to symptom monitoring *via* patient-reported outcomes have been published, although it may offer a valuable source of information and facilitate adherence.

Challenges are also faced when treating secondary hypothyroidism, including central hypothyroidism, in the elderly. Since TSH cannot be applied as a titration target, fT4 in the upper level of the reference range is normally recommended as target  (53, 67). However, no clinical evidence is available on how the cautious strategy regarding L-T4 replacement in elderly patients with primary hypothyroidism (a higher TSH) should be translated into their counterparts with secondary hypothyroidism. It seems prudent to aim for fT4 in the lower half of the reference range in older patients, paying attention to lipids and body mass index (68–70), and closely monitoring symptoms and signs of overtreatment. Randomized clinical trials targeting various fT4 ranges and evaluating other clinical measures of thyroid function and QoL are also in this situation highly warranted.

## Adherence and Risk of Overtreatment in Elderly Patients With Hypothyroidism

The limited QoL-impact of hypothyroidism and the associated subtle treatment effect experienced in elderly patients challenges treatment motivation and thus adherence. As mentioned above, polypharmacy, a high degree of co-morbidity, particularly cognitive co-morbidity, further challenge adherence. For the latter, the often-complex L-T4 regimen, with doses varying over weekdays to achieve optimal titration, may be a particular challenge. Polypharmacy also leads to difficulties obtaining ideal absorption; patients with e.g., dementia may have difficulty taking levothyroxine separate from other medications and in the fasting state, as generally recommended. Management strategies to counteract these obstacles may involve dosing boxes and possibly even weekly dosing. Efficacy of such action remains to be elucidated, as does e.g. a potentially useful dosing at bedtime, separate from other medication  (71). In case L-T4 tablet malabsorption is suspected, different formulation of L-T4 (e.g., liquid or gel) could be considered (57, 58).

**Figure 2:**
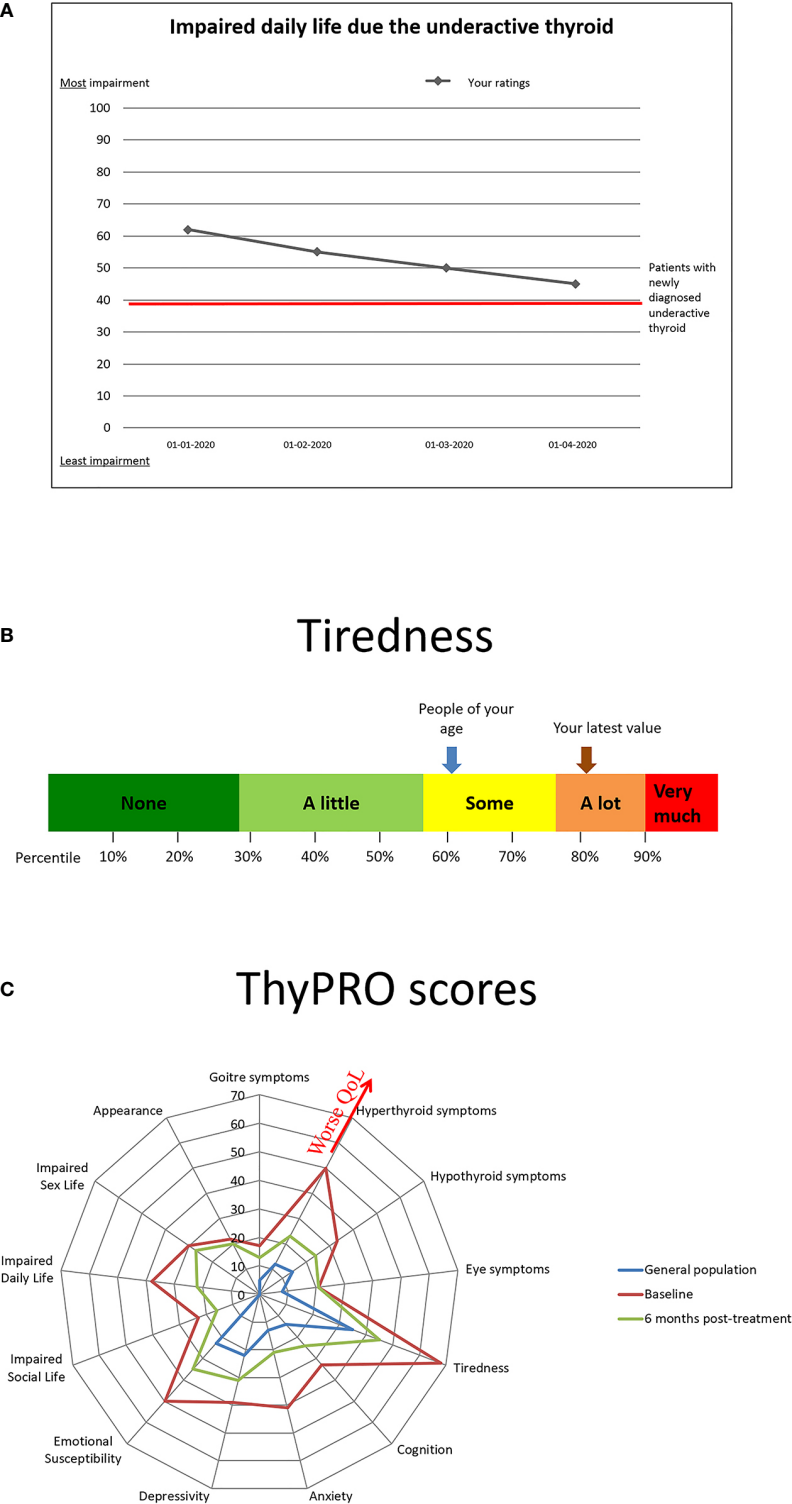
Examples of presentations of results from patient-reported outcomes recorded prior to a patient visit. **(A)** Patient-oriented presentation with reference to previous ratings. **(B)** Patient-oriented presentation with reference values as percentiles and general population reference. **(C)** Clinician-oriented multidimensional quality of life (QoL) presentation.

In other diseases, particularly within oncology and rheumatology, implementation of PROs as monitoring and communication tools has led to improved patient-clinician interaction and patient satisfaction (72). A groundbreaking study by Basch et al. showed improved management, QoL, morbidity, health care use and mortality, when implementing a systematic patient-reported symptom monitoring system among cancer patients (73). Unexpectedly, the effect was strongest in patients with the least resources and education. It is possible, that implementation of such a system, within the electronic health records of elderly patients with overt or subclinical hypothyroidism, would guide treatment decisions, including a decision to abstain from treatment of subclinical hypothyroidism in case of no recognizable patient-reported effect, improve treatment adherence and identify adverse effects. In practice, patients would complete a standardized, validated PRO prior to their appointment with their endocrinologist/physician, the results of which would be entered directly into the health record. In case the PRO results are presented in a comprehensible way, as e.g., illustrated in **Figures 2A, B**, it may form a useful communication tool between the patients and their health caretakers (74). As a tool for monitoring of and improving adherence to L-T4 treatment, the ThyPRO appears to be a relevant candidate  (75, 76), given its wide application and well-documented validity  (77). The multidimensional results of a ThyPRO completion is often displayed as a radar-plot, as in **Figure 2C**, but an optimal format for patient communication still remains to be established. Studies evaluating the effect of implementing PRO measures in clinical management of hypothyroidism among the elderly (or in any thyroid population) are still awaited.

The risk of overtreatment with L-T4 cannot be overemphasized especially in the elderly (9, 78–80). Five to 24 percent of all patients taking L-T4 develop iatrogenic thyrotoxicosis (9, 11, 78, 81–83), a proportion that is even higher in the elderly [up to 41% (79)]. Approximately half of the prevalent and incident low TSH events are related to overtreatment with L-T4, with the highest rates among older women (84). Overtreatment is associated not only with a suppressed TSH concentration but may also result in higher concentrations of fT4 compared with healthy controls (27). Thyroid hormones in excess are catabolic on the one hand while essential for stimulating the general basic metabolic rate (resting energy expenditure) on the other (85, 86). Overtreatment with L-T4 thus results in adverse effects due to acceleration of these physiological effects (85, 86). Consequently, overstimulating the catabolic metabolism is putting too high a pressure on the human organism which will eventually lead to loss of important and vital functions from failing productions of vital organ components.

Thus, suppressed TSH has in population studies consistently been associated with a higher mortality and other adverse effects compared to people with normal or even higher TSH concentrations (**Table 5**) (87–90). Apart from the higher mortality in patients with suppressed TSH the most important risks of overtreatment are those affecting the heart (50), the bones  (91), the brain (92) and muscle function (93). Many of the studies on the effect of a higher thyroid function than normal on the various organ risks, however, come from patients with endogenously elevated thyroid hormones and suppressed TSH, which can nevertheless be considered a surrogate for iatrogenic hyperthyroidism as described below.

**Table 5:** Major risks from overtreatment with levothyroxine of elderly patients with overt or subclinical hypothyroidism.

Cardiac arrythmias (atrial fibrillation or other tachyarrythmias)
Global decrease in cardiac physical performance
Progressive heart failure
Loss of bone mineral content progressing to osteoporosis
Progressive catabolic muscle loss progressing to muscle insufficiency
Other catabolic consequences such as loss of protein and vitamins and other substances
Cognitive disturbance progressing to premature dementia
Progressive impairment of quality of life
Premature death - most often cardiac

Older patients with low TSH and higher fT4 have a higher prevalence and incidence of atrial fibrillation compared with euthyroid subjects (94–98), >5-fold higher likelihood for the presence of atrial fibrillation in both patients with subclinical and overt hyperthyroidism (98). In addition, the serum fT_4_ concentration was independently associated with atrial fibrillation in euthyroid subjects 65 years and older (95) and old individuals with TSH in the lowest quartile and fT4 in the highest quartile of the normal range had an increased risk of atrial fibrillation (99). Finally, thyroid-cancer patients receiving TSH suppressive doses of L-T4 had increased risk of cardiovascular and all-cause mortality (100).

Most data on the skeletal effects of thyroid hormone excess support increased bone loss and risk of fractures in post-menopausal women and elderly men with thyrotoxicosis. Subclinical hyperthyroidism was also associated with greater annual bone loss at the femoral neck but not at the lumbar spine in prospective cohorts (101), while in euthyroid women with a history of Graves’ hyperthyroidism lumbar spine bone density was negatively associated with TSH receptor antibodies in post- but not premenopausal women (102). L-T4 treated women with low TSH concentrations lose bone mineral from the spine more rapidly compared with women without known thyroid disease (103), and TSH-suppressive therapy was associated with a significant bone loss at both the lumbar spine and hip in postmenopausal, but not in premenopausal, women  (91). Increased bone loss and risk of fracture was also found in euthyroid postmenopausal women with fT4 and/or fT3 levels within the upper normal range and in older adults with low TSH (104–106). The effect of current use of L-T4 treatment in elderly on the risk of fractures seems dose-related (107), particularly in women aged ≥65 years with osteoporosis (108). Additionally, the risk of a non-vertebral fracture was increased in euthyroid postmenopausal women with higher fT4 and/or fT3 (104). Recently, radiological vertebral fractures in women with differentiated thyroid carcinoma receiving post-surgical levothyroxine treatment were significantly and independently associated with TSH <1.0 mU/l, age of patients, duration of L-T4 therapy and densitometric diagnosis of osteoporosis at any skeletal site (109).

It is not very clear if overtreatment with L-T4 causes cognitive and psychiatric disturbances as well as an impairment of QoL, but endogenous thyrotoxicosis is well known to have the capability to result in these brain affections (110–112), and can likely be used as surrogate markers for L-T4 overtreatment. Prospective studies, however, are needed for further clarification of the long-term risk of brain dysfunction in cases of overtreatment with T4.

Thyrotoxicosis induces a reduction of muscle mass (113) and few studies in young or non-elderly subjects have demonstrated reduced muscle strength, which is restored after normalization of thyroid hormones (114–119). In newly diagnosed patients with Graves’ disease the hyperthyroidism was associated with impaired maximum muscle strength, performance, and balance (120). However, in older adults, subclinical hyperthyroidism was not associated with low muscle mass and/or strength (121–123), but the association between TSH and low muscle strength was found to be U-shaped (123). Nevertheless, data on the association between T3/fT3 and muscle mass are conflicting and some studies found negative (35, 123), while others positive associations (124–126), with some differences between men and women.

## Future Clinical Trials and Developments

Evidently, further documentation on several aspects of L-T4 treatment in elderly patients are warranted.

First of all, large randomized clinical trials among elderly patients with overt hypothyroidism targeting different TSH titration ranges are needed to guide future clinical practice.

Secondly, large randomized clinical trials evaluating safety and efficacy of L-T4 for subclinical hypothyroidism, ideally in several strata of TSH both at inclusion and as target, are needed for a personal medication approach to be evidence-based.

Thirdly, in both above trial settings, safety, including all aspects of risk of overreplacement should be investigated.

Fourthly, trials evaluating usefulness of implementing PRO measurements in L-T4 treatment and monitoring of elderly patients with both overt and mild/subclinical hypothyroidism should be performed, in order to evaluate, if such an approach provides value for clinicians and patients.

Finally, new biomarkers of thyroid function metabolism for monitoring efficacy of L-T4 therapy in the elderly should be sought for and, along with already existing candidates, evaluated properly in clinical studies.

## Author Contributions

All authors contributed to the idea, to the collection of information and references, writing of the manuscript and approval of the final manuscript. All authors contributed to the article and approved the submitted version.

## Funding

UF-R’s research salary was sponsored by The Kirsten and Freddy Johansen’s Fund.

## Conflict of Interest

The authors declare that the research was conducted in the absence of any commercial or financial relationships that could be construed as a potential conflict of interest.
